# Supplementary material for: Prevalence and Associated Factors for Purchasing Antibiotics Without a Prescription Among Patients in Rural South Africa: Implications for Addressing Antimicrobial Resistance
Source: Antibiotics (Basel). 2025 Dec 16;14(12):1273. doi: 10.3390/antibiotics14121273 (PMC12729307; doi:10.3390/antibiotics14121273)
Supplement: Supplementary file 1 [file antibiotics-14-01273-s001.zip › antibiotics-4010457-supplementary.pdf]

antibiotics MDPI

Supplementary Tables

Supplementary Table S1 – Continued concerns with the prescribing of antibiotics in primary care in South Africa

Author and year	Aim and methods	Key findings
Farley et al., 2018 [108]	• Objective is to research attitudes, knowledge and practices regarding antibiotics and ABR among primary care prescribers<br>• Cross sectional survey design with a self-administered questionnaire<br>• 264 prescribers completed the survey, with 98.3 % being physicians and 84.8 % practising in the private as opposed to the public sector	• 95.8% of those interviewed believed ABR is a major problem in South Africa<br>• 87.5% of interviewed prescribers expressed a desire for additional education regarding the appropriate use of antibiotics in primary care, with 96.2% also requesting data on local antibiotic resistance patterns to improve future prescribing<br>• Prescribers were also interested in the provision of STGs in different formats to improve future prescribing<br>• However, 66.5% of surveyed prescribers felt pressure from their patients to prescribe antibiotics for their presenting infectious disease irrespective whether antibiotics were needed
Gasson et al., 2018 [109]	• Assess current antibiotic prescribing habits among prescribers in PHCs and compare their prescribing against national STGs<br>• A retrospective review of antibiotic prescribing habits assessed alongside potential reasons for non-adherence to STGs<br>• 654 patient records were reviewed	• There was appreciable prescribing of antibiotics among patients attending these PHCs, with 68.7% of attending patients prescribed an antibiotic<br>• There were concerns with low adherence to STGs as adherence only occurred in 45.1% of prescriptions<br>• Principal reasons for STG non-adherence included: undocumented diagnoses - 30.5% of prescriptions, antibiotics not required including for self-limiting viral infections - 21.6%, incorrect doses of antibiotics prescribed - 12.9%, incorrect duration of antibiotic therapy prescribed - 9.5%, and incorrect treatment for the presenting infectious disease - 1.5%
Truter and Knoesen, 2018 [110]	• The objective is to determine antibiotic prescribing habits among primary care physicians using a self-administered questionnaire<br>• 16 community pharmacists participated in the study	• 81.3% of surveyed community pharmacists believed physicians were over-prescribing, including for viral infections, enhanced by patient pressure<br>• Amoxicillin /co-amoxiclav were the most prescribed antibiotics. This was followed by clarithromycin, ciprofloxacin and azithromycin<br>• Surveyed community pharmacists believed sinusitis and URTIs were the most common infectious diseases for which antibiotics were prescribed
van Hecke et al., 2019 [111]	• The objective was to determine the perceptions of clinicians working in PHCs concerning their prescribing of antibiotic prescribing for acute coughs and UTIs alongside their experiences concerning point-of-care testing	• Antibiotic prescribing decisions among participating HCPs in PHCs were typically influenced by a number of factors. These including their clinical assessment of presenting patients, their comorbidities and perceptions regarding the expectations of their patients<br>• There were observed difficulties in the communication between prescribers and patients, which often hampered efforts to explain non-antibiotic management strategies to patients including for viral infections<br>• In view of this, participating clinicians were typically positive towards current and future point-of-care testing,

Author and year	Aim and methods	Key findings
	• Qualitative interviews were undertaken among 23 prescribers	especially for viral infections, to improve evidence-based antibiotic prescribing<br>• Prescribers though had concerns with current resources and workflow issues influencing the uptake and use of such tests as part of their routine care of patients presenting with infectious diseases
Balliram et al., 2021 [112]	• The aim was to assess the knowledge, attitudes and practices of doctors, pharmacists and nurses regarding antimicrobials, AMR and AMS practices<br>• A national online survey was conducted among doctors, pharmacists and nurses	• 96.4% of doctors believed AMR was a severe global threat, with 96.6% also believing AMR is a significant problem in South Africa<br>• Having said this, only 37.70% of surveyed doctors felt ≤ 50% confidence in their knowledge regarding antimicrobials, AMR and AMS<br>• However, 94.9% of surveyed doctors believed antibiotics were not effective against viral infections (vs. e.g. 75.3% for nurses) and 99.1% that common colds are caused by viruses (vs. e.g. 90.2% nurses).<br>• 91.61% of participating HCPs believed the overuse of antimicrobials was the greatest contributor to AMR, followed by patient pressure (75.26%) and non-adherence to prescribed treatments (73.26%)<br>• Overall, 80.1% of participating HCPs expressed a need for more education and training on antimicrobial use, AMR, and AMS<br>• Participating doctors saw educational campaigns (91.22%), use of STGs (84.72%), and improved infection control measures (66.31%), as important strategies to combat AMR.
Govender et al., 2021 [113]	• The aim was to evaluate the use and implementation of the STGs/EML among prescribers (nurses) at a public tertiary institution and associated PHC facilities<br>• A mixed method approach was used - including evaluating patient records and undertaking interviews using a structured questionnaire	• 41% of surveyed nurses had access to the latest STG/EML<br>• All the participating nurses stated they often/ sometimes refer to the STG/EML when managing patients<br>• However, only 41% of surveyed nurses stated they had access to the latest STG/EML<br>• There was a 59.7% adherence rate for prescriptions to the STG/EML.<br>• Having said this, 94.9% of surveyed nurses requested training on the use of STGs/EML to improve their future prescribing which included antibiotics/ infectious diseases as most prescribers had not received any formal training on its use
Alabi et al, 2022 [114]	• The objective was to assess the appropriateness of antibiotic prescribing among practicing GPs in the private sector<br>• This included an analysis of antibiotic prescriptions (188,141) among 174,889 patients, with appropriateness based on ICD-10 classification and	• 92.9% of patients were prescribed one antibiotic by primary care GPs when attending their clinics, with 7.1 % prescribed two or more antibiotics<br>• Penicillins were the most prescribed antibiotics (40.7%) of all antibiotics prescribed - followed by macrolides (16.8%) and cephalosporins (15.7% - all generations combined)<br>• 46.1% of all diagnoses made included diseases of the respiratory system<br>• Only 8.8% of all the prescriptions were subsequently deemed as appropriate and 32.0% potentially appropriate<br>• 45.4% of prescriptions were deemed inappropriate and 13.8% could not be assessed due to a lack of specific codes/

Author and year	Aim and methods	Key findings
	whether an antibiotic was deemed warranted or not	contained unlisted codes/ or contained unclear descriptions in the prescription
De Vries et al., 2022 [115]	• The objective was to evaluate the impact of a multidisciplinary audit and feedback AMS intervention to improve future antibiotic prescribing<br>• The AMS included monthly feedback meetings at 13 PHCs using 10 prescriptions randomly selected for peer review<br>• Antibiotic prescriptions were subsequently scored for adherence to 7 key measures including antibiotic choices vs. recommendations in STGs<br>• Antibiotic utilisation patterns were also assessed	• There was suboptimal adherence to the current STGs at the start of the study at only 11%<br>• Encouragingly, adherence increased to 53% over a 2-year period<br>• However, adherence to STGs was significantly lower in the winter and spring - concurrent with higher antibiotic prescribing/ consumption. This potentially reflects inappropriate antibiotic prescribing for acute viral ARIs during these months<br>• Only 19% of prescriptions were correct in the first 6 months. This rose to a mean of 47% correct prescriptions in the last 6 months of the study ($p<0.001$) following active interventions.<br>• Overall, the AMS was associated with a 19.3% decrease in antibiotic consumption during the study period
Guma et al., 2022 [116]	• The objective was to assess current antibiotic empiric prescribing rates among private GPs for their patients attending with ARIs and associated key factors<br>• A semi-structured web-based questionnaire was used	• 209 GPs took part in the survey<br>• 55.5% of surveyed GPs admitted to prescribing antibiotics empirically for patients with ARIs more than 70% of the time - primarily for symptom relief and the prevention of complications<br>• Encouragingly, GPs with more experience and working alone were slightly less likely to prescribe antibiotics empirically<br>• Key factors significantly associated with empiric prescribing of antibiotics were workload/time pressures, diagnostic uncertainty and the use of a formulary
Keuler et al., 2022 [117]	• The aim was to assess the treatment of UTIs in PHCs and determine their compliance with current STGs/EML via a retrospective review of medical records of patients diagnosed with UTIs<br>• Overall, 6 PHCs took part - involving 401 UTIs	• Antibiotics were prescribed in all male and 98.5% of females with uncomplicated UTIs and in 98.3% of those with complicated UTIs<br>• Nitrofurantoin was prescribed to the majority of patients with UTIs (57.1%), followed by ciprofloxacin (39.7%) - nitrofurantoin appropriately selected in 75.0% of patients with uncomplicated UTIs<br>• In patients with complicated cases, compliance to STGs/ EML was higher with ciprofloxacin (44.4%) vs. nitrofurantoin (25.6%)<br>• Overall compliance with STGs was greater for patients with uncomplicated (61.5%) vs. complicated UTIs (52.9%), with failure to comply with STGs mostly due to inappropriate antibiotic selection for complicated UTIs and the duration of prescribed antibiotics

Author and year	Aim and methods	Key findings
Blaauw and Lagarde 2023 [118]	• The objective was to assess prescribing practices for young and healthy SPs presenting to PHCs with viral bronchitis including both private (99 SPs) and public PHCs (102 SPs)<br>• Alongside this, 125 providers (across both sectors) were also interviewed face-to-face using a structured questionnaire	• Antibiotics were recommended in 72.6% of SP consultations, higher in the public sector (78.4%) vs. private sector (66.7%) - enhanced by perceived patient pressure<br>• These high rates were despite 84% of prescribers knowing the SP case was likely to be a viral infection (88% in the private sector vs. 77% in the public sector) and 58% knowing that antibiotics would not hasten recovery (40% public vs. 68% private; p=0.002)<br>• 47% of prescribers in public PHCs thought patients would not come back if they did not prescribe an antibiotic – higher in the private sector at 72% (p=0.008) - despite SPs not demanding antibiotics<br>• Encouragingly, antibiotic prescribing rates were lower in both sectors (20% lower) in a previous study when HCPs were explicitly told by their patients that they did not want antibiotics for their infection unless they were really necessary
Van Hecke et al, 2024 [119]	• The objective was to assess the impact of a pharmacist-prescriber partnership to review antibiotic prescribing in public PHCs<br>• 457 patients with acute coughs were enrolled at 5 PHCs.	• 84% of patients enrolled in the review were prescribed an antibiotic for their acute cough<br>• The most prescribed antibiotics for these patients were amoxicillin (63%), co-amoxiclav (13%) and phenoxymethylpenicillin (6%), with a diagnosis of 'community-acquired pneumonia' the principal indication (35%)<br>• There was also a significant proportion of patients prescribed an antibiotic for 'acute cough' which needs addressing with future AMS activities including community pharmacists to reduce AMR
Wieters et al., 2024 [120]	• The aim was to assess antibiotic use by WHO AWaRe classification among patients visiting healthcare facilities in 4 African countries including South Africa<br>• The infectious diseases surveyed included acute febrile disease of unknown cause (AFDUC), gastro-intestinal (GI) infections and RTIs	• Out of the 36.8% of patients across the 4 countries stating they had taken antibiotics in the previous 10 days, 41.5% were prescribed for RTIs, 30.3% for AFDUC and 22.6% for GI infections. There were similar rates for RTIs in South Africa at 41.4% and AFDUC at 27.8%<br>• The most common antibiotic prescribed in the study was ceftriaxone (31.7% of antibiotics prescribed – lower in South Africa)<br>• Among patients with RTIs, the prescribing of ampicillin was highest in South Africa (22.8%) – with ceftriaxone at 15.0%
Chigome et al, 2025 [61]	• A point prevalence survey was undertaken among PHCs in 2 Provinces in South Africa and repeated<br>• This was part of a larger study	• Data for 615 patients were recorded in the PPS study with the most common symptoms for antibiotics being a genital discharge (21.8%), painful urination (18.4%), acute cough (17.7%), and a sore throat (13.5%), with patients potentially having more than one symptom<br>• At least one antibiotic was prescribed to 87.0% of attending patients<br>• Access antibiotics accounted for 53.4% of all antibiotics prescribed, with 46.6% from the Watch group. Ceftriaxone

Author and year	Aim and methods	Key findings
		(29.7%), amoxicillin (29.4%) and azithromycin (28.4%) were the most frequently prescribed antibiotics<br>• Overall considerable concerns with current prescribing practices among PHCs in South Africa
Maluleke et al, 2025 [60]	• 75.7% (128/169) of operational pharmacies in this rural province participated in this questionnaire-based study, with independent pharmacies representing the majority of these (60.9%)<br>• 78.3% response rate with 106 pharmacists and 207 pharmacist assistants taking part	• Antibiotics accounted for 47.9% of all medicines dispensed among the surveyed community pharmacies with penicillins the most dispensed antibiotic (41.1% of the total).<br>• 47.2% of antibiotics dispensed included cephalosporins, macrolides and fluoroquinolones – typically Watch antibiotics.<br>• STIs (33.5%) and URTIs (25.8%) were the most frequent indications for antibiotics<br>• Overall, limited dispensing of antibiotics without a prescription - estimated at only 8.6% of the total volume of antibiotics dispensed<br>• Encouragingly, 98.1% of community pharmacists and 97.6% of pharmacist assistants indicated they always or mostly offered symptomatic relief before suggesting/ dispensing antibiotics without a prescription to patients with typically self-limiting conditions
Sono et al, 2025 [59]	• Pilot study assessing patients' understanding of key terms including antibiotics when leaving community pharmacies<br>• Patients were also questioned if leaving with antibiotics whether these were prescribed or dispensed	• 11 patients took part in the pilot study to assess their understanding of key terms such as antibiotics and AMR using their own language<br>• Among patients dispensed an antibiotic with a prescription – the majority (66.7%) were for URTIs with 33.3% for STIs<br>• STIs were also the most prevalent indication when antibiotics were dispensed without a prescription - with limited dispensing of antibiotics without a prescription for URTIs (12.5%)

NB: ABR = Antibiotic Resistance; AMR = Antimicrobial Resistance; AMS = Antimicrobial Stewardship; ARI = Acute Respiratory Infection; AWaRe = Access, Watch and Reserve [21]; EML = Essential Medicines List; GPs = General Practitioners; PHCs = Primary Healthcare Clinics; RTIs = Respiratory Tract Infections; SPs = Simulated Patients; STGs = Standard Treatment guidelines; STIs = Sexually Transmitted Infections; URTIs = Upper Respiratory Tract Infections; UTIs = Urinary Tract Infection

Supplementary Table S2 – Examples of the influence of patients and carers in primary care across LMICs regarding antibiotic use and their lack of knowledge

Country, authors and year	Key findings
**Influence of patients on antibiotic utilisation**	
Ethiopia - Altaye et al., 2024 [101]	Key factors increasing the prescribing of antibiotics in PHC centres included a lack of respect for prescribers among patients coupled with pressure from patients on prescribers to prescribe antibiotics - exacerbated by limited knowledge and awareness regarding antibiotics
India - Nair et al., 2019 [121]	Over 88% of surveyed physicians in PHC centres reported prescribing antibiotics for viral infections - enhanced by patient demands, with similar rates among informal providers
Jordan - Orubu et al., 2022 [48]	Among participating prescribers in PHC centres, 65% reported pressure to prescribe antibiotics based on a number of factors including the patients' condition (78%); perceived patient demand (60%); and the perceived need to provide rapid relief (47%)
Malawi - MacPherson et al., 2021 [122]	High rates of prescribing of antibiotics in primary care exacerbated by limited time with each patient and fear of criticism for not prescribing medicines including antibiotics during the consultation
Nepal - Acharya et al., 2021 [49]	High levels of dispensing of antibiotics without a prescription were influenced by pressure from patients especially when patients specifically asked for 'an antibiotic' – with 'customer satisfaction' as the most important factor motivating community pharmacists in Nepal
Tanzania - Ndaki et al., 2023 [102]	High levels of dispensing of antibiotics exacerbated by a number of factors including pressure/demands from patients coupled with business orientation/ financial gain of dispensing outlets
**Knowledge and attitudes of patients regarding antibiotics and AMR**	
Bangladesh - Islam et al., 2024 [123]	• There were concerns that an appreciable proportion of surveyed parents had a lack of knowledge with recognizing basic antibiotics, e.g. 63% and 56% respectively of parents did not know that amoxicillin and azithromycin were antibiotics<br>• In addition, 63% believed that antibiotics could be used to treat colds as well as improve a fever coupled with a cold, and 27% reused the same antibiotic for similar symptoms
Egypt - Alsayed et al., 2022 [50]	• Whilst 54.5% of surveyed adults believed antibiotics was problem, 58.8% believed antibiotics were effective against sore throats and 57.6% believed they were effective against colds, coughs and nasal congestion<br>• 48.3% also believed antibiotics were effective against fevers and 39.4% against viral infections generally
Ethiopia - Muhummed et al., 2024 [51]	89.5% of participating patients believed that antibiotics can be used to treat watery diarrhoea, 71% when they have a fever, 68.2% when they have a common cold and 57.7% for general viral infections
Ghana - Otieku et al., 2023 [124]	Among 800 participants, 71% disagreed or disagreed to some extent that AMR may lead to reduced productivity/indirect costs, 87% disagreeing that AMR increases provider costs and 59% that AMR increases costs for carers, patients/societal costs
Ghana - Vicar et al., 2023 [103]	• Despite claiming good knowledge concerning the use of antibiotics and AMR, a significant proportion of the 600 surveyed households held misconceptions about antibiotics and their use<br>• Specifically, 77.0% of household personnel believed antibiotics can cure influenza and 42.4% believed antibiotics can treat headaches or coughs
Malawi - Machongo et al., 2022 [125]	Caregivers of children under 5 typically self-medicated their children with antibiotics to treat coughs, sore throats and diarrhoea
Nigeria - Isah et al., 2023 [104]	Despite some knowledge of AMR, among 964 participating patients, 50.9% believed that antibiotics can relieve fever and pain, 22.1% believed antibiotics can cure their cold/sore throat faster and 27.9% that using leftover antibiotics was permissible for another cold or similar symptoms
Pakistan - Gillani et al., 2021 [126]	• Among 2106 members of the public, 60.2% had generally low levels of knowledge regarding antibiotic use

	• This was illustrated by 35.4% believing antibiotics could cure viral infections, with only 47.8% disagreeing that antibiotics are effective against colds and influenza. In addition, only 46.3% agreed that it is not necessary to treat a cold with antibiotics
Zambia - Kampamba et al., 2024 [127]	Overall variable knowledge of antibiotics and AMR among surveyed patients - 58.2% stated they had taken an antibiotic for a common cold, 70.5% had used leftover antibiotics from previous courses and 74.0% had bought their antibiotics without a prescription

NB: ARI = Acute respiratory infection; AMR = Antimicrobial resistance; HCPs = Healthcare professionals; PHC = Primary healthcare

Supplementary Table S3**.** Details of antibiotics dispensed with or without a prescription by pharmacy category

	Number (%) of patients who received an antibiotic		
**Pharmacy category**	**With a prescription***	**Without a prescription***	**Total****
Chain pharmacies	27 (100)	0	27 (10.7)
Independent pharmacies	13 (8.1)	148 (91.9)	161 (63.6)
Franchise pharmacies	14 (21.5)	51 (78.5)	65 (25.7)
**Total**	**54 (21.3)**	**199 (78.7)**	**253**

NB: *Row percentages; **Column percentages

Supplementary Table S4: Antibiotic items dispensed with and without a prescription distributed by their AWaRe category

	**Number (%) of antibiotics dispensed without a prescription**			
AWaRe Category	Observed	41	171	212
Access	% within row	19.3%	80.7%	100%
	% within column	63.1%	55.0%	56.4%
Watch	Observed	24	140	160
	% within row	14.6%	85.4%	100%
	% within column	36.9%	45.0%	43.9%
Reserve	Observed	65	311	376
	% within row	17.3%	82.7%	100%
	% within column	100%	100%	100%

NB: AWaRe – Access, Watch and Reserve [21]

Supplementary Table S5 - Impact of initiates involving community pharmacists being allowed to prescribed selected antibiotics for agreed indications

**Country, Author and Year**	**Aims and Methods**	**Key Findings**
Canada – Beahm et al., 2018 [75]	• Prospective registry trial among 750 patients with UTIs treated at 39 community pharmacies<br>• Pharmacists were permitted to prescribe antibiotics, modify current prescriptions for antibiotics, provide education only or refer the patients to a physician	• 88.9% of patients achieved a clinical cure<br>• Adverse events were reported by 7.2% of patients, with 88.9% continuing their medication – most were gastrointestinal-related and transient<br>• Overall, very high levels of satisfaction for the care they received from community pharmacists as well as for trust and accessibility

Canada – Sanyal et al, 2019 [128]	• The objective was to compare the costs and outcomes of community pharmacist-initiated management of UTIs vs. physicians or emergency management<br>• UTI cure rates and utilities for the study were derived from published studies	• Community pharmacist-initiated management had the lowest cost ($72.47) vs. physician ($141.53) or emergency management ($368.16)<br>• Outcomes (quality-adjusted-life-months) were comparable across the 3 different strategies<br>• If only 25% of patients with uncomplicated UTI were managed by community pharmacists over the next 5 years – it was estimated that the resultant net total savings would be approximately $51 million
New Zealand – Gauld et al., 2017 [129]	• Assess the impact of community pharmacists undergone training to supply trimethoprim to women with uncomplicated cystitis<br>• Women were invited to self-complete a questionnaire, with national prescribing data extracted for antibiotic use	• Baseline data were provided by 139 pharmacies with 120 providing post-implementation data, with prescriptions for cystitis primarily being trimethoprim<br>• Antibiotic use did not increase post-implementation<br>• Trimethoprim use by specially trained pharmacists within strict criteria appeared to have little overall effect on total antibiotic use, i.e. no increase was seen with this development
United Kingdom - Booth et al., 2013 [130]	• Compare the care pathway of patients with UTI symptoms attending GP services with those receiving management, including trimethoprim<br>• Trimethoprim could be provided by community pharmacists under a patient group direction (PGD) initiative for moderate-to-severe uncomplicated UTIs	• Data on 153 patients were recorded<br>• 97 patients were treated by GPs with a prescription<br>• 56 patients presented directly to pharmacist with symptoms suggestive of UTIs - 41 subsequently received trimethoprim via a PGD and 15 ha symptomatic management<br>• Overall, there was demand and support from patients for access to antibiotics for UTIs from community pharmacists
United Kingdom – Hind 2018 [131]	• Assessment and treatment of uncomplicated lower UTIs in adult women by community pharmacists<br>• Audit of 349 anonymized patient treatment assessment forms	• Community pharmacists saw patients quickly - around 90% of patients were seen in less than 10 minutes<br>• Some patients commented this process was quicker and easier than obtaining an appointment and seeing a GP<br>• Trimethoprim was provided to 299 patients with 21 patients rereferred as they could not be treated by the pharmacist<br>• Pharmacists were able to treat UTIs appropriately including the correct use of trimethoprim<br>• Re-treatment levels were less than seen in similar audits with GPs
United Kingdom – Stewart et al., 2018 [132]	• A 'Pharmacy First' scheme was introduced in one locality in Scotland for the management of patients with UTIs, impetigo and an exacerbation of COPD<br>• Appropriately trained pharmacists supplied a limited range of prescription medicines with a quantitative evaluation undertaken of the service	• I75 pharmacies and 55 GP practices participated with 1189 cases managed principally for UTIs (75.4%)<br>• Of all cases, 77.9% were prescribed medication by the pharmacist, 9.1% were only given advice and 16.7% were referred to their GP<br>• Overall, patients were very satisfied with the service - most frequently citing the 'quick and efficient' access to treatment and a 'professional service'<br>• 67% of GPs (67%) and 59% of reception staff found the service useful – principally because it reduced pressure on GP appointments
United Kingdom –	• Evaluate the effectiveness and uptake of a lower UTI test-and-treat service for women	• 764 women who presented to 23 community pharmacies<br>• Lower UTIs were seen as likely in 372/496 (75.0%) women - most of whom purchased antibiotics on the same day

Thornley et al., 2020 [74]	presenting with UTIs in a community pharmacy<br>• Antibiotics could be prescribed if deemed necessary by the pharmacist	• If the service was not there – ¾ would have visited their GP at some point with more than 1/3rd using self-care<br>• Overall, a community pharmacy-led UTI test-and-treat service for women with urinary symptoms provided accessible and timely care, which was welcomed
Multiple countries including Australia, Canada, and the UK - Jebara et al., 2018 [133]	• Critically appraise, synthesize and present available evidence on the views and experiences of key stakeholders on community pharmacist prescribing<br>• Present perceived facilitators and barriers for global implementation.	• 65 studies were identified, mostly from the UK (n = 34) and Australia (n = 13), with most studies reporting pharmacists' perspectives, with fewer on patients' perspectives<br>• The principal benefits of pharmacist prescribing were (1) ease of patient access; (2) improved patient outcomes; (3) better use of pharmacists' skills and knowledge (4) improved job satisfaction among participating community pharmacists, and (5) reduced physician workload
Multiple countries including Canada and the UK – Wu et al., 2021 [73]	• A systematic review was conducted to characterize existing studies regarding key issues surrounding community pharmacist prescribing of systemic antimicrobials<br>• 3793 articles were identified with 14 meeting the inclusion criteria	• Antibiotics were being prescribing for UTIs, acute pharyngitis and cold sores<br>• Prescribing was associated with (1) high rates of clinical improvement (4 studies), low rates of retreatment and adverse effects (3 studies) and decreased health care use (7 studies).<br>• Patients were highly satisfied with the situation as they could access care sooner/ or more easily<br>• Pharmacists' interventions reduced unnecessary prescribing for acute pharyngitis (2 studies) and increased the appropriateness of prescribing for UTIs (3 studies).
Multiple countries including Canada, New Zealand and the UK – Swart et al, 2024 [76]	• To systematically gather, assess, and synthesize peer-reviewed published papers regarding the management of uncomplicated UTIs by community pharmacists in women aged 16–65 years<br>• Qualitative studies and non-primary studies were excluded	• 2129 records were assessed in the review with high self-reported cure rates of UTIs of between 84 and 89%<br>• Encouragingly, referral rates to physicians were low at approximately 7% were reported<br>• However, there were no randomized controlled trials and the papers were of variable quality limiting the conclusions

NB: UTI = Urinary Tract Infection

Supplementary Table S6 – Examples of successful ASPs among all key stakeholder groups across LMICs including African countries

Country, author, year	Brief details and outcome
Prescribers	
Eswatini, Ness et al 2021 [134]	• The ASP involved implementing STGs in Eswatini coupled with education of prescribers<br>• Following the ASP, there was a significant decrease in the proportion of visits to PHCs where antibiotics were prescribed ($p < 0.001$)<br>• In addition, the extent of antibiotic prescribing for incorrect indications decreased from 20.4% in the initial period to 10.31% and 10.2% in subsequent study periods<br>• Alongside this, the prescribing of incorrect doses/duration of antibiotics prescribed decreased from 10.47% in the initial period to 7.37% and 3.1% in subsequent periods<br>• Overall, all prescribers taking part in the study believed the introduction of STGs positively influenced their future prescribing of antibiotics

South Africa, De Vries et al 2022 [115]	• The instigation of multidisciplinary audit and monthly feedback meetings among prescribers in 13 PHCs improved future antibiotic prescribing<br>• The mean overall level of adherence to current guidelines increased from a baseline of 11% to 53% over a 2-year period<br>• Alongside this, there was an appreciable rise in correct prescriptions (p<0.001) - rising from a mean of 19% in the first 6 months (mean) to 47% in the last 6 months of the study. Accompanying this was a 19.3% decrease in antibiotic use during the study period
South Africa, Van Hecke et al 2025 [135]	• Introduction of a group messaging service to provide feedback to prescribers alongside auditing of their antibiotic prescriptions improved prescribing<br>• This combined approached improved the proportion of pharmacy-dispensed antibiotics that agreed with local STGs in terms of the quality of prescribing to 95% of all antibiotic prescriptions
Zanzibar, Hadley et al 2019 [136]	• An ASP was undertaken to assess a performance-based financing model to improve antibiotic prescribing using quality indicators based on adherence to current STGs – with active and control groups<br>• The ASP resulted in a fall in the number of patients not treated in accordance with the STGs in the active group to 2%, 6% and 5% in 2014, 2015 and 2016, respectively, versus an increase from 25% (2013) to 31% (2014) and 22% (2015, 2016) in the control groups<br>• Overall, rigorous monitoring of prescriber habits is needed for any sustained reduction in unnecessary antibiotic prescribing among PHCs
Pharmacists – also including education among patients	
Egypt, Kandeel et al., 2019 [137]	• An ASP was undertaken to raise community pharmacists' awareness concerning appropriate use of antibiotics for patients presenting with ARIs through educational campaigns, with educational courses/ campaigns subsequently undertaken among 596 pharmacists<br>• Mean knowledge scores regarding the judicious use of antibiotics increased from 3.3 ± 0.9 to 4.0 ± 1.2, with attitude scores regarding unnecessary dispensing of antibiotics for colds (to preserve their effectiveness) also improving<br>• Pharmacists' attitudes concerning not overusing/ dispensing antibiotics to prevent resistant bacteria also improved following the ASP
Indonesia - Ferdiana et al., 2024 [138]	• A 7-month ASP was undertaken among 80 community pharmacies which comprised: (a) online educational sessions for community pharmacists; (b) awareness campaign targeting patients; (c) peer visits to community pharmacies; and (d) pharmacy/ pharmacist certification<br>• Overall, there was a significant reduction following the ASP in the % of antibiotics dispensed for URTIs, UTIs and childhood diarrhoea<br>• Overall, antibiotic dispensing significantly decreased from 82.3% of consultations in the control group to 55.4% in the active group<br>• The pre-post difference in the dispensing of antibiotics without a prescription in active group was 20.9% vs. only 2.3% in the control group (p value <0.001).<br>• Overall, the dispensing of antibiotics without a prescription was less likely in the active group (OR=0.19; 95% CI 0.09 to 0.43)
Kenya, Mukokinya et al., 2018 [139]; Opanga et al., 2021 [140]; Kimathi et al., 2022 [141]; Sono et al, 2023 [34]	• Training of community pharmacists at Universities in Kenya regarding antibiotics and AMR reduced inappropriate dispensing of antibiotics without a prescription for self-limiting conditions such as ARIs. This is reflected by:<br>o No antibiotics were dispensed without a prescription to patients presenting with ARIs, including common colds or influenza, at community pharmacies allied to the University of Nairobi [34,139]<br>o This contrasts with a high degree of self-medication with antibiotics among patients presenting with acute infections at community pharmacies/ drugs

	stores during the COVID-19 pandemic in Kenya (23.6%) - Kimathi et al., 2022 [141]. This compares with the findings from 6 pharmacies allied to the University of Nairobi where no pharmacists dispensed antibiotics to patients with COVID-19 without a prescription. Instead, symptomatic relief/ advice was offered instead [140], enhanced by improved knowledge of antibiotics and AMR.
Namibia - Kamati et al., 2019 [142]; Kibuule et al., 2019 [143]; Sono et al, 2023 [34]	• Multiple activities in Namibia including activities among the health authorities in Namibia to ban the purchasing of antibiotics without a prescription, combined with regular monitoring of community pharmacies, alongside ongoing training of pharmacists at the University regarding antibiotics and AMR as well as generally good access to PHC centres, limited inappropriate dispensing of antibiotics without a prescription among community pharmacies in the country<br>• This was reflected by:<br>o No dispensing of antibiotics without a prescription to children under 5 presenting with ARIs – treatment was typically symptomatic and included paracetamol, cold/ flu medicines, and decongestants<br>o Training alongside the provision of alternative treatments were given to patients visiting community pharmacies at the start of the COVID-19 pandemic to prevent/ symptomatically treat COVID-19 - with no dispensing of antibiotics without a prescription
Republic of Srpska - Marković-Peković et al, 2017 [144]; Bojanić et al, 2018 [145]	• Multiple activities have been undertaken in the Republic to improve antibiotic use and reduce AMR. These include (a) education of pharmacists in universities and post-qualification regarding the appropriate management of infectious diseases, including for patients with self-limiting viral infections, (b) development and distribution of guidelines to improve the management of the most frequent diseases/ conditions seen in everyday practice in community pharmacies including for infectious diseases, (c) training of community pharmacists to improve their communication skills with patients and (d) greater enforcement of regulations banning the dispensing of antibiotics without a prescription in the Republic alongside fines - Euro500–1500 for pharmacy directors/ Euro500–750 for pharmacy technicians<br>• These multiple activities resulted in:<br>o Dispensing of antibiotics without a prescription to patients presenting with infectious diseases, including with self-limiting viral infections, decreased from 58% to 18.5% of surveyed pharmacies.<br>o OTC therapy offered to simulated patients in 72.3% of pharmacies visited - up from 67.2% in previous studies, with symptomatic relief including analgesics and antihistamines, throat and nasal sprays, decongestants, and oral expectorants, typically offered instead, with significantly fewer pharmacies dispensing antibiotics without a prescription where symptomatic relief was more appropriate<br>o The most common reason for not dispensing antibiotics to simulated patients in the recent study was that these could not be dispensed without a prescription.
Thailand – Arparsrithongsagul et al 2015 [146]	Activities included:<br>• Principally education involving a multidisciplinary intervention among grocery stores in a rural province in Thailand<br>• Trained community leaders were used to reduce the extent and number of antibiotics currently in participating village grocery stores<br>• There were 87% fewer antibiotics available post-intervention compared with pre-intervention

	• This compared with grocery stores in the control group where there was only an 8% reduction in antibiotic availability between the two time periods
Uganda - Bagonza et al, 2021 [147]	• An ASP was instigated which involved peer-supervision among drug sellers (keeping registers) concerning the appropriate treatment of non-bloody diarrhoea and pneumonia among children under 5 years of age.<br>• The ASP resulted in the proportion of children appropriately treated for pneumonia symptoms increasing by 10.84% in the intervention group<br>• In addition, the proportion of children with non-bloody diarrhoea appropriately treated was 4% higher in the intervention group

NB: ASP = Antimicrobial Stewardship Programme; PHCs = Primary Healthcare Clinics; RTIs = Respiratory Tract Infections; SPs = Simulated Patients; STGs = Standard Treatment guidelines; STIs = Sexually Transmitted Infections; URTIs = Upper Respiratory Tract Infections; UTIs = Urinary Tract Infection

Supplementary Table S7 – Patient Questionnaire

Date: __________ **Participant no: _______**

Greet the patient and invite them to participate in the survey. Provide the patient with the participant information sheet or read it for them. Upon agreement to participate in the survey, obtain signed informed consent.

1. Age	_____ years	Prefer not to disclose age			
2. Biological sex assigned at birth	Male	Female	Prefer not to answer		
**3. Home language**	Xitsonga	Tshivenda	Sepedi	English	Other (Specify)
**4. Educational level**	No education	Primary school completed	High school completed	ABET certificate	College certificate
	Diploma	Bachelor's degree	Honours degree	Master's degree	Doctorate

**5. Were you dispensed or sold any medication at the pharmacy today?**	Yes	No	
**6. Did you have a prescription from a doctor?**	Yes	No	
**7. Does the medication include any antibiotics?**	Yes	No	Don't know
**8. For what condition did you visit the pharmacy?**			
**How many items were dispensed/sold to you?**	Yes	No	***Note: If no antibiotics were dispensed, end the interview and thank the patient for their time***

**9. If antibiotics were dispensed, ask to have a look at the antibiotics dispensed and enter the details**	**Antibiotics dispensed**	**Other medication**
	1.	1.
	2.	2.
	3.	3.
	4.	4

**10. What were the antibiotics indicated for?**	URTI (Upper respiratory tract infection)		SSTI (Skin and soft tissue infection)	
	STI (Sexually transmitted disease)		UTI (Urinary tract infection)	
	Other (Please specify)			

**11. Who were the antibiotics for?**	Adult	Child	Both
**12. If the medication is for a child under 12, are you the parent or guardian?**	Parent	Guardian	Other (specify)
**13. If the medication is for a child, how old are they?**	____________ years/months		

**14. If any antibiotics were obtained without a prescription, what were the reasons?**	Clinic too far	Long waiting times
	No money to see a doctor	Used the same antibiotics before
	Lack of knowledge	Don't know
	Pharmacist recommended them	Patient insisted on an antibiotic
	Other (Please specify)	

Thank the patient for their time and participation
